# Impact of cervical spine immobilization on clinical outcomes in traumatic brain injury patients according to prehospital mean arterial pressure: A multinational and multicenter observational study

**DOI:** 10.1097/MD.0000000000032849

**Published:** 2023-02-17

**Authors:** Eujene Jung, Young Sun Ro, Hyun Ho Ryu, Sang Do Shin

**Affiliations:** a Department of Emergency Medicine, Chonnam National University Hospital, Gwangju, Korea; b Department of Emergency Medicine, Seoul National University Hospital, Seoul, Korea; c Department of Emergency Medicine, Chonnam National University Hospital, Gwangju, KoreaMedicine, Chonnam National University, Gwangju, Korea; d Department of Emergency Medicine, Seoul National University, Seoul, Korea.

**Keywords:** cervical spine immobilization, outcome, traumatic brain injury

## Abstract

Cervical spine immobilization (CSI) has been considered an essential part of first aid management after severe trauma; however, the routine use of CSI for traumatic brain injury (TBI) patients is a matter of debate. The purpose of our study was to analyze the effect of CSI on the clinical outcomes of TBI patients and to analyze whether this effect depends on the prehospital mean arterial pressure (MAP) This was a prospective multi-national, multi-center cohort study using Pan-Asian trauma outcome study registry in Asian-Pacific, conducted on adult trauma patients. The main exposure was the implementation of CSI before hospital arrival. The main outcome was poor functional recovery at hospital discharge measured by the modified rankin scale. We performed multilevel logistic regression analysis to estimated the effect size of CSI for study outcomes. Interaction analysis between CSI and MAP on study outcomes were also conducted. CSI for TBI patients is significantly associated with an increased poor functional outcome (adjusted odd ratio, 95% confidence intervals: 1.23 [1.03 – 1.44]). The association of CSI with poor functional outcomes was maintained only in patients with decreased prehospital MAP (1.38 [1.14 – 1.56]), but not in patients with normal MAP (1.12 [0.93 – 1.24]) (*P* for interaction < .05). Routine use of CSI for patients with TBI, but without cervical spine injury, is associated with poor functional outcomes, but is significant only when the MAP, measured at the scene, was decreased.

## 1. Introduction

Prehospital care of traumatic brain injury (TBI) patients plays a crucial role in the quality of hospital care as it can significantly affect subsequent care outcomes. The aim of prehospital care is to minimize secondary injuries while optimizing the well-being of patients.^[1]^

Cervical spinal cord injury is a severe and life-threatening complication of trauma, which may be accompanied by TBI.^[2]^ If the cervical spine is not properly immobilized at the prehospital stage, secondary complications are likely to occur. Hence, preventing the risk of cervical spine injury by adopting methods such as cervical spine immobilization (CSI) is an understandable concern for emergency medical service paramedics.^[3,4]^

CSI has been used for more than 30 years and is considered an essential part of first aid management after severe trauma, especially in unconscious patients. This is because it was believed that immobilizing the spine would be the best way to prevent spinal cord injury following a traumatic injury.^[5,6]^

However, cervical spine injury is relatively rare in trauma patients, occurring in 2% to 4% of patients admitted to hospitals due to traumatic injury.^[3]^ Furthermore, routine use of CSI may compromise the team ability to maintain an airway and poses a risk for both patients and paramedics by delaying extraction from a dangerous area. Moreover, it can be very uncomfortable for the patient, especially if the immobilization is prolonged.^[5,7]^

In TBI patients, CSI has been shown to increase intracranial pressure (ICP) and consequently, decreases cerebral perfusion pressure (CPP), which could be harmful for survival and neurological outcomes.^[8–10]^ This rise in ICP has been found to be, on average, ~4.5 mm Hg.^[8,11]^

We hypothesized that the effect of CSI to decrease CPP by increasing ICP would vary depending on the mean arterial pressure (MAP) – the lower the MAP, the more severe the harmful effect of CSI would be.

Therefore, the purpose of our study was to analyze the effect of the routine use of CSI on the survival and neurological outcomes of TBI patients, and to analyze whether this effect depends on the MAP measured in the field.

## 2. Materials and methods

### 2.1. Study design, setting, and data sources

This was a prospective multicenter study using the Pan-Asian trauma outcome study (PATOS) registry in the Asia-Pacific region.^[12]^

PATOS is an Asia-Pacific clinical research network with a multicenter trauma registry. It was established in 2013 to collect trauma data from the Asia-Pacific region; 85 centers from the following countries participated in this network on a voluntary basis: China, India, Japan, Laos, Malaysia, Philippines, Korea, Singapore, Taiwan, Thailand, UAE, and Vietnam. The age-standardized mortality rate per 100,000 injured persons varies from 25.8 in Japan to as high as 91.4 in India.^[13]^ There are differences in the trauma care systems within the various countries in the Asia-Pacific region. The emergency medical service (EMS) performance in some countries in the Asian-Pacific region remains below international standards,^[14]^ and many countries are experiencing strains due to limited resources. The EMS operation is hospital-based in 5 countries, fire-based in 4 countries, volunteer-based in 2 countries, and public health-based in 1 country. The top level of EMS providers is Physician in 5 countries, emergency medical technician (EMT)-intermediate in 4 countries, EMT-basic, EMT-paramedic, and multiple levels in 1 country each.^[12]^ Moreover, trauma registration is not established in most low- and middle-income countries; if such registries even exist, they are often incomplete and rudimentary.^[15]^

The PATOS collects information on demographic findings, injury epidemiology, prehospital factors, hospital factors, and the outcomes of patients with injury. Prehospital data are collected from ambulance run sheets or EMS dispatch records. Hospital records and patient outcome data are collected from the hospital medical records. To maintain standardized and consistent data quality, training modules were developed to educate all personnel involved in registering data. All data are entered via an electronic data capture system. Definitions and coding instructions of all variables are laid out in a data dictionary, which is distributed to all participating sites. Data are then collated via an electronic data capture system (see http://epatos.org). The aggregated data have to be cleaned and managed by the PATOS data quality management committee to address invalid and/or incomplete entries. All sites must respond to the PATOS Data quality management committee within 2 weeks of receiving data verification requests.

### 2.2. Study population

Our study population comprised adult trauma patients, over 18 years of age, who visited the participating hospitals between January 2015 and December 2020. Patients with unknown information on MAP, CSI, and clinical outcomes at hospital discharge were excluded.

### 2.3. Main outcomes

The primary outcome measure was poor functional recovery at hospital discharge measured by the modified Rankin Scale. Poor functional recovery was defined by modified Rankin Scale scores of 4 (moderately severe disability), 5 (severe disability), and 6 (death). The secondary outcome was mortality at hospital discharge.

### 2.4. Variables and measurements

The main exposure of our study was the implementation of CSI before emergency department arrival. We collected data on the patients’ demographics (country, age, and sex), injury characteristics (intentionality, place of injury, activity at the time of injury, and mechanism of injury), prehospital variables [MAP = (2 × diastolic blood pressure + systolic blood pressure)/3, advanced airway, and fluid resuscitation], emergency department and hospital variables (injury area, systolic blood pressure, heart rate, and glasgow coma scale), and clinical outcomes at hospital discharge^[12]^

### 2.5. Statistical analysis

We compared the characteristics of the patients according to CSI implementation before hospital arrival using the chi-square test for categorical variables and the Wilcoxon rank-sum test for non-parametrically distributed continuous variables.

Multivariable logistic regression analyses were performed to estimate the effect size of CSI for poor functional recovery and mortality at hospital discharge after adjusting for potential confounders. Adjusted odds ratios with 95% confidence intervals were calculated. In addition, subgroup analysis was conducted to further clarify the effect of CSI on the clinical outcome of brain injury. Finally, the effect of the interaction between CSI and MAP on the study outcomes was analyzed to investigate whether the effect of CSI implementation on clinical outcomes of TBI patients was modified according to prehospital MAP.

All statistical analyses were performed using SAS version 9.4 (SAS institute Inc., Cary, NC). All *P* values were 2-tailed, and *P* < .05 was considered statistically significant.

## 3. Results

### 3.1. Demographic findings

**Figure 1.**
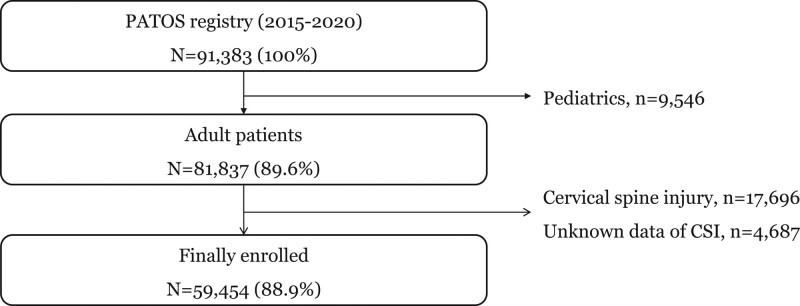
Study populations.

From 2015 to 2020 in the PATOS registry, a total of 59,472 trauma patients were finally enrolled. We excluded patients who did not use EMS (n = 43,325), were under 18 years old (n = 9546), with cervical spine injury (n = 10,694), and with unknown data on CSI (n = 4678) (Fig. 1).

The characteristics of the study population according to CSI are shown in Table 1. Of the 59,454 trauma patients, CSI was applied to 25.0% (14,822/59,454). For all trauma patients, the proportion of in-hospital mortality was 5.6% (3310/59,454): 6.3% (941/14,822) in the patients with CSI, and 5.3% (2369/44,632) in the patients without CSI (*P* < .01). The rate of poor functional outcomes was 21.7% (12,906/59,454): 22.5% (3332/14,822) in the patients with CSI and 21.5% (9574/44,632) in the patients without CSI (*P* = .14). Of the 18,754 patients with TBI, CSI was applied to 39.9% (7490/18,754). For the TBI patients, the proportion of in-hospital mortality was 5.1% (962/18,754): 5.4% (401/7490) in the patients with CSI and 5.0% (561/11,264) in the patients without CSI (*P* = .04). The rate of poor functional outcomes was 19.8% (3716/18,754): 20.1% (1508/7490) in the patients with CSI and 19.6% (2208/11,264) in the patients without CSI (*P* = .09).

**Table 1:** Characteristics of all patients and traumatic brain injury patients according to the cervical spine immobilization.

Variables	All patients				Patients with TBI			
		Cervical spine immobilization				Cervical spine immobilization		
	Total	Yes	No	*P* value	Total	Yes	No	*P* value
All	59,454 (100.0)	14,822 (100.0)	44,632 (100.0)		18,754 (100.0)	7490 (100.0)	11,264 (100.0)	
Country				< .01				< .01
Korea	44,552 (74.9)	10,428 (70.4)	34,124 (76.5)		14,027 (74.8)	5424 (72.4)	8603 (76.4)	
Malaysia	12,671 (21.3)	3362 (22.7)	9309 (20.9)		3719 (19.8)	1491 (19.9)	2228 (19.8)	
Other	2231 (3.8)	1032 (7.0)	1199 (2.7)		1008 (5.4)	575 (7.7)	433 (3.8)	
Age (years)				< .01				< .01
18–65	43,326 (72.9)	11,456 (77.3)	31,870 (71.4)		13,075 (69.7)	5521 (73.7)	7554 (67.1)	
65-	16,128 (27.1)	3366 (22.7)	12,762 (28.6)		5679 (30.3)	1969 (26.3)	3710 (32.9)	
Sex, female	21,800 (36.7)	4178 (28.2)	17,622 (39.5)	< .01	6097 (32.5)	2155 (28.8)	3942 (35.0)	< .01
Intentionality, yes	7486 (12.6)	622 (4.2)	6864 (15.4)	< .01	1655 (8.8)	292 (3.9)	1363 (12.1)	< .01
Place of injury				< .01				< .01
Home	16,511 (27.8)	1819 (12.3)	14,692 (32.9)		4354 (23.2)	1035 (13.8)	3319 (29.5)	
Street	29,665 (49.9)	10,433 (70.4)	19,232 (43.1)		10,199 (54.4)	5021 (67.0)	5178 (46.0)	
Other	13,278 (22.3)	2570 (17.3)	10,708 (24.0)		4201 (22.4)	1434 (19.1)	2767 (24.6)	
Mechanism				< .01				< .01
Traffic accident	22,595 (38.0)	9774 (65.9)	12,821 (28.7)		7716 (41.1)	4579 (61.1)	3137 (27.8)	
Fall	19,481 (32.8)	3790 (25.6)	15,691 (35.2)		7476 (39.9)	2305 (30.8)	5171 (45.9)	
Other	17,378 (29.2)	1258 (8.5)	16,120 (36.1)		3562 (19.0)	606 (8.1)	2956 (26.2)	
Acute alcohol use, yes	12,619 (21.2)	2853 (19.2)	9766 (21.9)	< .01	5104 (27.2)	1761 (23.5)	3343 (29.7)	< .01
Prehospital vital signs								
Mean arterial pressure (median, IQR)	98 (90–106)	97 (89–106)	98 (90–106)	.07	99 (90–107)	98 (89–107)	99 (91–107)	.12
HR (median, IQR)	87 (76–98)	87 (77–96)	88 (76–98)	.14	88 (77–98)	88 (77–97)	89 (77–99)	.11
Mental change, yes	12,302 (20.7)	4599 (31.0)	7703 (17.3)	< .01	5420 (28.9)	3110 (41.5)	2310 (20.5)	< .01
Prehospital treatment				< .01				< .01
Advanced airway	500 (0.8)	401 (2.7)	99 (0.2)		223 (1.2)	196 (2.6)	27 (0.2)	
Fluid resuscitation	3809 (6.4)	2328 (15.7)	1481 (3.3)		1634 (8.7)	1249 (16.7)	385 (3.4)	
Severity of trauma (NISS)				< .01				< .01
1–8	37,669 (63.4)	7701 (52.0)	29,968 (67.1)		11,850 (63.2)	3585 (47.9)	8265 (73.4)	
9–15	7634 (12.8)	2545 (17.2)	5089 (11.4)		2402 (12.8)	1282 (17.1)	1120 (9.9)	
16–24	2481 (4.2)	1461 (9.9)	1020 (2.3)		1268 (6.8)	866 (11.6)	402 (3.6)	
25–75	11,670 (19.6)	3115 (21.0)	8555 (19.2)		3234 (17.2)	1757 (23.5)	1477 (13.1)	
Study outcomes								
Poor functional recovery	12,906 (21.7)	3332 (22.5)	9574 (21.5)	.14	3716 (19.8)	1508 (20.1)	2208 (19.6)	.09
In-hospital mortality	3310 (5.6)	941 (6.3)	2369 (5.3)	< .01	962 (5.1)	401 (5.4)	561 (5.0)	.04

HR = heart rate, IQR = interquartile range, NISS = new injury severity score, TBI = traumatic brain injury.

### 3.2. Main outcomes

In multivariable logistic regression analysis, CSI was not associated with in-hospital mortality or poor functional outcome in total trauma patients. In patients with TBI, although CSI was not associated with in-hospital mortality, it was significantly associated with an increased rate of poor functional outcomes (1.23 [1.03–1.44]) after adjusting for potential confounders (Table 2).

**Table 2:** Multivariable logistic regression analysis on study outcomes categorized by cervical spine immobilization.

	Total	Outcome		Model 1	Model 2
	N	N	%		
In-hospital mortality					
Total patients	59,454	3310	5.6		
Immobilization (-)	44,632	2369	5.3	ref.	ref.
Immobilization (+)	14,822	941	6.3	1.34 (0.79–2.30)	1.32 (0.77–2.27)
Patients with TBI	18,754	962	5.1		
Immobilization (-)	11,264	561	5.0	ref.	ref.
immobilization (+)	7490	401	5.4	1.14 (0.88–1.32)	1.16 (0.91–1.34)
Poor functional outcome					
Total patients	59,454	12,906	21.7		
Immobilization (-)	44,632	9574	21.5	ref.	ref.
Immobilization (+)	14,822	3332	22.5	1.18 (0.88–1.45)	1.19 (0.91–1.45)
Patients with TBI	18,754	3716	19.8		
Immobilization (-)	11,264	2208	19.6	ref.	ref.
Immobilization (+)	7490	1508	20.1	1.19 (0.98–1.40)	1.23 (1.03–1.44)

Model 1: adjusted for age, sex, intentionality, place and mechanism of injury, acute alcohol use.

Model 2: adjusted for variables in Model 1, mental change, and severity of trauma.

TBI = traumatic brain injury.

### 3.3. Interaction analysis

Interaction analysis was used to assess whether study outcomes of CSI varied according to prehospital MAP. The odds ratios (ORs) for poor functional outcomes of TBI patients differed according to MAP (*P* < .01). The association of CSI with poor functional outcomes was maintained only in patients with decreased prehospital MAP [1.38 (1.14–1.56), but not in patients with normal MAP (1.12 (0.93–1.24)] (Table 3).

**Table 3:** Interaction analysis between the cervical spine immobilization and prehospital mean arterial pressure on study outcome.

	Immobilization (-)	Immobilization (+)	
	aOR (95% CI)	aOR (95% CI)	*P* for interaction
In-hospital mortality			
Total patients			.88
Normal MAP	reference	1.45 (0.74–1.88)	
Decreased MAP	reference	1.33 (0.92–1.75)	
Patients with TBI			.21
Normal MAP	reference	1.29 (0.98–1.45)	
Decreased MAP	reference	1.22 (0.88–1.63)	
Poor Functional outcome			
Total patients			.98
Normal MAP	reference	1.17 (0.95–1.46)	
Decreased MAP	reference	1.20 (0.88–1.50)	
Patients with TBI			< .01
Normal MAP	reference	1.12 (0.93–1.24)	
Decreased MAP	reference	1.38 (1.14–1.56)	

aOR = adjusted odds ratio, CI = confidence interval, MAP = mean arterial pressure, TBI = traumatic brain injury.

## 4. Discussion

This prospective, multinational, multicenter study using a registry of injury in the Asia-Pacific region examined whether CSI was associated with poor clinical outcomes when routinely applied to trauma patients without cervical spine injury. We found that in patients without cervical spine injury but with TBI, CSI was associated with poor functional outcomes, but only in patients with low prehospital MAP.

Advanced trauma life support was started in 1978 and has been adopted by over 50 countries.^[16]^ Routine CSI is one of the key recommendations of advanced trauma life support, which was developed because it was believed that immobilization of the cervical spine would be the best way to prevent spinal cord injury following trauma.^[6]^

However, cervical spine injury is relatively rare in trauma patients.^[3]^ In a large retrospective study in the US, 740 (2.3%) patients among 32,117 trauma patients who applied routine CSI before spine evaluation had cervical spine injury.^[3]^ In another study in the US, cervical spine injury was observed in 61 (4.6%) of 1331 trauma patients who did not apply CSI; however, none developed permanent neurological deficits.^[17]^ Therefore, it has been suggested that the risk of cervical nerve injury due to improper cervical immobilization may be overestimated.^[18]^

In a multinational study comparing a hospital in New Mexico, where CSI is routinely used for all trauma patients, and a Malaysian hospital, without any routine prehospital CSI, there was less overall neurological disability in the Malaysian patients.^[19]^ The authors suggest that movements during transport are unlikely generated a large amount to cause further injury to the spine and spinal cord.^[19]^ In addition, to our knowledge, no human patient study has demonstrated clear benefit from the application of rigid CSI in patients with cervical injury during transport.

In TBI patients, ICP may have already been increased due to hypoxia, hypocarbia, intracranial hematoma, and cerebral edema, while cerebral blood flow could be decreased due to hypotension and loss of cerebral autoregulation.^[20,21]^ In addition, increased jugular venous pressure due to CSI may exacerbate the decrease in CPP accompanied by an increased ICP. A meta-analysis comparing the ICP values during CSI and after CSI removal reported an overall ICP decrease of approximately 3 mm Hg after CSI removal.^[22]^ Changes of 3 mm Hg could have significant effects, and a small rise in jugular venous pressure could also have disastrous consequences on cerebral blood flow.^[23]^ This could explain the results of our study that routine use CSI in TBI patients is related to poor functional outcomes.

However, it is difficult to explain why harmful effects of CSI on TBI patients were not observed in patients with normal MAP in this study. One possible explanation is that patients with normal MAP can maintain high CPP despite ICP elevation due to CSI, but patients with decreased MAP may experience an increase in ICP, resulting in a lower CPP.

In our study, the application of CSI to TBI patients did not have any association with in-hospital mortality but had a positive association with poor functional outcomes only when the MAP measured at the prehospital stage was decreased. To our knowledge, this is the first study to analyze the interaction between CSI and MAP with respect to the clinical outcomes of TBI patients. The results of our study reinforce the hypothesis proposed in previous studies that CSI worsens the clinical outcome of TBI patients by increasing ICP, thereby reducing CPP.

Our finding that CSI is associated with poor functional outcomes in TBI patients, especially in patients with decreased MAP at the scene, may provide the theoretical basis for proposing additional guidelines for the use of CSI in patients with TBI, but without cervical spine injury.

Our study has several limitations. First, our study is a multinational, multicenter study, and different types of CSI are applied in each country and region, which may affect the study results. Second, the explanation of our finding that CSI increases ICP and consequently lowers CPP is only a hypothesis as our study did not measure the ICP of the TBI patients. Third, because blood pressure measurements can be high due to the pain and agitation of patients immediately after trauma,^[24,25]^ it is possible that the MAP calculated by measurements taken in the prehospital stage overestimated the patients’ blood pressure in our study. Fourth, the investigators of the PATOS registry were not blinded to the study hypothesis, which could have led to biased data collection from the data gatherers. Finally, as the study design was not a randomized controlled trial, there may be potential biases that were not controlled.

## 5. Conclusions

Routine use of CSI for patients with TBI, but without cervical spine injury, is associated with poor functional outcomes, but is significant only when the MAP, measured at the scene, was decreased.

## Author contributions

**Conceptualization:** Young Sun Ro, Hyun Ho Ryu.

**Data curation:** Hyun Ho Ryu.

**Formal analysis:** Eujene Jung, Hyun Ho Ryu.

**Investigation:** Eujene Jung.

**Methodology:** Sang Do Shin.

**Project administration:** Eujene Jung, Sang Do Shin.

**Resources:** Young Sun Ro.

**Software:** Eujene Jung, Young Sun Ro, Sang Do Shin.

**Supervision:** Young Sun Ro, Hyun Ho Ryu, Sang Do Shin.

**Visualization:** Eujene Jung.

**Writing – original draft:** Eujene Jung.

**Writing – review & editing:** Hyun Ho Ryu.
